# Supplementary material for: Neural basis of corruption in power-holders
Source: eLife. 2021 Mar 24;10:e63922. doi: 10.7554/eLife.63922 (PMC7990503; doi:10.7554/eLife.63922)
Supplement: Supplementary file 1. [file elife-63922-supp1.docx]

**Supplementary Information for**

**Neural basis of corruption in power-holders**

Yang Hu^1,2†^ , Chen Hu^3,4^, Edmund Derrington^2,5^,

Brice Corgnet^6^, Chen Qu^1*^, Jean-Claude Dreher^2,5*^

^1^ Center for Studies of Psychological Application, School of Psychology, South China Normal University, Guangzhou, China

^2^ Laboratory of Neuroeconomics, Institut des Sciences Cognitives Marc Jeannerod, CNRS, Lyon, France

^3^ Motivation, Brain & Behavior (MBB) Team, Institut du Cerveau et Moelle Epiniere, Hôpital de la Pitié-Salpêtrière, Paris, France

^4^ Sorbonne Université, Paris, France

^5^ Université Claude Bernard Lyon 1, Lyon, France

^6^ EmLyon, Ecully, France

^*^Correspondence: [fondest@163.com](mailto:fondest@163.com) (C.Q.)

^†^Present Address: Key Laboratory of Applied Brain and Cognitive Sciences, School of Business and Management, Shanghai International Studies University, Shanghai, China

**This PDF file includes:**

Supplementary File 1a to 1k

**Supplementary File**

**Supplementary File 1a.** Output of fixed effects in mixed-effect logistic regressions predicting acceptance decisions

|  | All trials^a^ | Solo^b^ | Dyad^b^ |
| --- | --- | --- | --- |
|  | *b*  (SE) | *b*  (SE) | *b*  (SE) |
| intercept | 3.55^***^  (0.36) | 3.76^***^  (0.37) | 3.12^***^  (0.30) |
| scenario | -0.18  (0.18) |  |  |
| proposer’s conduct | -1.82^***^  (0.41) | -1.91^***^  (0.41) | -2.03^**^  (0.39) |
| scenario × proposer’s conduct | -0.40^*^  (0.18) |  |  |
| offer proportion^c^ | 10.83^***^  (0.37) | 12.08^***^  (0.59) | 9.90^***^  (0.48) |
| larger payoff in the reported option^c^ | 0.002  (0.004) | -0.002  (0.005) | 0.006  (0.005) |
|  |  |  |  |
| AIC | 3535.9 | 1709.7 | 1943.8 |
| BIC | 3622.1 | 1757.3 | 1991.3 |
| *N* (Observation) | 5616 | 2808 | 2808 |
| *N* (Participant) | 39 | 39 | 39 |

Note: ^a^ We included the by-subject and by-run random-effect intercept. In addition, we also included scenario and proposer’s conduct as the by-subject random-effect slopes.

^b^ We included the by-subject and by-run random-effect intercept. In addition, we also included proposer’s conduct as the by-subject random-effect slope.

^c^ These variables were grand mean-centered before putting into the regression model.

Reference levels were set as follows: scenario = Solo, proposer’s conduct = Control. Table also shows goodness-of-fit statistics: AIC = Akaike Information Criterion, BIC = Bayesian Information Criterion. Significance: ^*^p < 0.05, ^**^p < 0.01, ^***^p < 0.001.

**Supplementary File 1b.** Bayesian model evidence

| Model | No. of Free Parameters | LOOIC |
| --- | --- | --- |
| 1 | 4 | 3110.472 |
| 2 | 5 | 3094.812 |
| 3 | 5 | 3079.893 |
| 4 | 6 | 3051.952 |
| 5* | 6 | 3033.296 |
| 6 | 9 | 5596.171 |
| 7 | 13 | 4681.949 |

Note: Lower LOOIC scores indicate better models. *refers to the winning model (M5). Abbreviations: LOOIC: leave-one-out information criterion.

**Supplementary File 1c.** Bivariate correlation between parameter estimates of the individual-level posterior mean based on the winning model

|  | β_PH_ | β_P_ | θ | ω | γ |
| --- | --- | --- | --- | --- | --- |
| β_P_ | -0.19 |  |  |  |  |
| θ | -0.42** | 0.18 |  |  |  |
| ω | -0.46** | 0.17 | 0.26 |  |  |
| γ | -0.25 | 0.17 | 0.65*** | 0.34* |  |
| τ | -0.12 | 0.12 | 0.05 | -0.19 | -0.01 |

Note: *Pearson* correlation coefficients are reported in the table.

Significance: ^*^*p* < 0.05, ^**^*p* < 0.01, ^***^*p* < 0.001

**Supplementary File 1d.** Descriptive summary for the reaction time (RT; in ms) of making decisions

|  |  | SC | SB | DC | DB |
| --- | --- | --- | --- | --- | --- |
| accept | Mean  (SD) | 1714.1  (619.1) | 2109.3  (805.8) | 1859.6  (728.0) | 2269.5  (1031.6) |
|  | *N* | 39 | 38 | 39 | 38 |
| reject | Mean  (SD) | 2467.6  (1059.0) | 2511.7  (1223.4) | 2535.8  (1240.7) | 2618.8  (1091.6) |
|  | *N* | 34 | 38 | 36 | 38 |

Note: we first calculated the individual-level mean decision time in terms of specific decisions in each experimental condition, then we calculated the group-level mean (± SD) based on the individual mean; Due to individual difference in decisions, the sample size (i.e., N) for each dilemma of specific decisions is different. Abbreviations: SC: Solo Control, SB: Solo Bribe, DC: Dyad Control, DB: Dyad Bribe.

**Supplementary File 1e.** Output of fixed effects in mixed-effect linear regressions predicting log-transformed reaction time (RT) of making decisions

|  | all trials^a^ | acceptance^b^ | rejection^c^ |
| --- | --- | --- | --- |
|  | *b*  (SE) | *b*  (SE) | *b*  (SE) |
| intercept | 7.29^**^  (0.13) | 7.27^**^  (0.15) | 7.64^***^  (0.09) |
| decision | 0.26^***^  (0.05) |  |  |
| scenario | 0.07^**^  (0.02) | 0.07^***^  (0.02) | 0.03  (0.04) |
| proposer’s conduct | 0.20^***^  (0.04) | 0.20^***^  (0.04) | 0.004  (0.04) |
| decision × scenario | -0.04  (0.05) |  |  |
| decision × proposer’s conduct | -0.22^***^  (0.06) |  |  |
| scenario × proposer’s conduct | -0.003  (0.03) | -0.01  (0.03) | 0.01  (0.05) |
| decision × scenario × proposer’s conduct | 0.05  (0.06) |  |  |
| offer proportion^d^ | -0.29^***^  (0.03) | -0.44^***^  (0.04) | 0.74^***^  (0.08) |
| larger payoff in the reported option^d^ | -0.002^**^  (0.0005) | -0.002^*^  (0.0007) | -0.001  (0.0009) |
|  |  |  |  |
| AIC | 7325.4 | 5302.4 | 1888.8 |
| BIC | 7643.8 | 5372.0 | 1963.0 |
| N (Observation) | 5616 | 4138 | 1478 |
| N (Participant) | 39 | 39 | 38 |

Note: ^a^ We included the by-subject and by-run random-effect intercept. In addition, we also included decision, scenario, proposer’s conduct and their interactions (i.e., three 2-way interaction terms and one 3-way interaction) as the by-subject random-effect slopes.

^b^ We included the by-subject and by-run random-effect intercept. In addition, we also included proposer’s conduct as the by-subject random-effect slope.

^c^ We included the by-subject and by-run random-effect intercept. In addition, we also included scenario and proposer’s conduct as the by-subject random-effect slope.

^d^ These variables were grand mean-centered before putting into the regression model.

Reference levels were set as follows: decision = accept, scenario = Solo, proposer’s conduct = Control. Table also shows goodness-of-fit statistics: AIC = Akaike Information Criterion, BIC = Bayesian Information Criterion. Significance: ^**^p < 0.01, ^***^p < 0.001.

**Supplementary File 1f.** Brain regions that encode payoff-related parametric modulators (PM; N = 39; GLM1a, GLM1a-s, GLM1b, GLM1b-s)

| Brain Regions | Hemisphere | Cluster Size | MNI | | | BA | T-value |
| --- | --- | --- | --- | --- | --- | --- | --- |
|  |  |  | x | y | z |  |  |
| **Regions encoding the expected profits due to bribe-taking**  **depending on conditions (GLM1a)** | | | | | | | |
| **Expected Personal Gains**  ***Main effect of scenario:*** |  |  |  |  |  |  |  |
| ***Dyad - Solo*** |  |  |  |  |  |  |  |
| PoCG/SmG/IPL | L | 140 | -60 | -26 | 36 | 2/3/4 | 3.89 |
| IPL/SmG/PoCG^*^ | R | 220 | 54 | -30 | 30 | 40 | 3.76 |
| ***Solo - Dyad*** |  |  |  |  |  |  |  |
| No activated cluster |  |  |  |  |  |  |  |
| ***Main effect of proposer’s conduct:*** |  |  |  |  |  |  |  |
| ***Bribe - Control*** |  |  |  |  |  |  |  |
| Ins/STG | L | 106 | -38 | 8 | -2 | 13/38 | 3.83 |
| ***Control - Bribe*** |  |  |  |  |  |  |  |
| No activated cluster |  |  |  |  |  |  |  |
| ***Interaction effect:*** |  |  |  |  |  |  |  |
| ***(DB - DC) – (SB - SC)*** |  |  |  |  |  |  |  |
| No activated cluster |  |  |  |  |  |  |  |
| ***(SB - SC) – (DB - DC)*** |  |  |  |  |  |  |  |
| No activated cluster |  |  |  |  |  |  |  |
|  |  |  |  |  |  |  |  |
| **Expected Gains for the Proposer** |  |  |  |  |  |  |  |
| No activated cluster in any of the  contrast above |  |  |  |  |  |  |  |
|  |  |  |  |  |  |  |  |
| **Regions encoding the expected profits due to bribe-taking**  **(pooled all conditions; GLM1a-s)** | | | | | | | |
| **Expected Personal Gains** |  |  |  |  |  |  |  |
| ***Positive modulation:*** |  |  |  |  |  |  |  |
| dmPFC/SFG/MFG^***^ | R | 534 | 6 | 56 | 36 | 8/9 | 4.51 |
| MeFG/ACC^*^ | R | 227 | 6 | 46 | 10 | 10/32 | 4.65 |
| vmPFC/MeFG/ACC^***^ | B | 572 | 4 | 52 | -16 | 10/11/32 | 4.20 |
| AG/MTG | L | 143 | -54 | -66 | 32 | 39 | 4.37 |
| AG/SmG/IPL/MTG/STG^***^ | R | 478 | 50 | -66 | 22 | 39/40 | 4.58 |
| MTG/ITG^**^ | R | 395 | 64 | -12 | -16 | 21 | 5.19 |
| Cerebellum | B | 163 | -2 | -50 | -30 |  | 4.20 |
| Cerebellum | L | 148 | -16 | -80 | -34 |  | 4.17 |
| ***Negative modulation*** |  |  |  |  |  |  |  |
| No activated cluster |  |  |  |  |  |  |  |
|  |  |  |  |  |  |  |  |
| **Expected Gains for the Proposer** |  |  |  |  |  |  |  |
| ***Positive modulation*** |  |  |  |  |  |  |  |
| No activated cluster |  |  |  |  |  |  |  |
| ***Negative modulation*** |  |  |  |  |  |  |  |
| LG/MOG/SOG | L | 232 | 8 | -94 | 14 | 17 | 4.12 |
| Cuneus | R | 154 | -16 | -90 | -2 | 17/18 | 4.47 |
|  |  |  |  |  |  |  |  |
| **Regions encoding the expected financial loss to the third party**  **(only in the DB condition; GLM1b)** | | | | | | | |
| ***Positive modulation*** |  |  |  |  |  |  |  |
| ACC/MeFG^**^ | B | 459 | 8 | 42 | 26 | 9/10/32 | 4.69 |
| MTG/STG/PHG^**^ | R | 426 | 54 | -48 | -4 | 13/22 | 4.70 |
| MTG/STG/SmG/IPL | R | 127 | 46 | -52 | 24 | 39 | 4.32 |
| Cerebellum^*^ | B | 284 | 14 | -54 | -30 |  | 4.22 |
| ***Negative modulation*** |  |  |  |  |  |  |  |
| No activated cluster |  |  |  |  |  |  |  |
|  |  |  |  |  |  |  |  |
| **Regions encoding the expected financial loss to the third party**  **(only in the DB condition; control analysis; GLM1b-s)** | | | | | | | |
| ***Positive modulation*** |  |  |  |  |  |  |  |
| ACC/MeFG^**^ | B | 553 | 8 | 42 | 26 | 9/10/32 | 4.76 |
| MTG/STG/PHG^**^ | R | 435 | 54 | -48 | -4 | 13/22 | 4.63 |
| IFG | R | 125 | 52 | 30 | 8 | 45/46 | 4.11 |
| AG/SmG/MTG/STG | R | 153 | 46 | -52 | 24 | 39 | 4.40 |
| Cerebellum | B | 207 | 14 | -54 | -30 |  | 4.25 |
| ***Negative modulation*** |  |  |  |  |  |  |  |
| No activated cluster |  |  |  |  |  |  |  |

Note: Regions shown here met the uncorrected voxel-level threshold of p < 0.001 with k = 100.

With the above uncorrected threshold as the cluster-defining threshold, clusters met the Family-Wise Error corrected cluster-level (cl-FWE) threshold were marked: ^*^p < 0.05, ^**^p < 0.01, ^***^p < 0.001

Coordinates shown here were based on Montreal Neurological Institute (MNI) coordinate system. Abbreviations: SC: Solo Control, SB: Solo Bribe, DC: Dyad Control, DB: Dyad Bribe; B: bilateral, L: left, R: right, BA: Brodmann Area; ACC: anterior cingulate cortex, AG: angular gyrus, dmPFC: dorsomedial prefrontal cortex, IFG: inferior frontal gyrus, Ins: insula, IPL: inferior parietal lobule, LG: lingual gyrus, MeFG: medial frontal gyrus, MFG: middle frontal gyrus, MOG: middle occipital gyrus, MTG: middle temporal gyrus, PHG: parahippocampal gyrus, PoCG: post-central gyrus, SFG: superior frontal gyrus, SmG: supramarginal gyrus, SOG: superior occipital gyrus, STG: superior temporal gyrus, vmPFC: ventromedial prefrontal cortex.

**Supplementary File 1g.** Brain regions that encode relative subjective values (SV; N = 39, GLM2a and GLM2b)

| Brain Regions | Hemisphere | Cluster Size | MNI | | | BA | T-value |
| --- | --- | --- | --- | --- | --- | --- | --- |
|  |  |  | x | y | z |  |  |
| **Regions encoding the relative SV**  **in Bribe and Control conditions (GLM2a)** | | | | | | | |
| **Control Conditions**  **(pooled trials in SC and DC conditions)** |  |  |  |  |  |  |  |
| ***Positive Modulation*** |  |  |  |  |  |  |  |
| MTG/ITG | R | 156 | 56 | -12 | -22 | 20/21 | 4.84 |
| AG/MTG/IPL | L | 147 | -42 | -68 | 30 | 39 | 4.25 |
| MFG/SFG | L | 120 | -20 | 24 | 40 | 8 | 3.99 |
| ***Negative Modulation*** |  |  |  |  |  |  |  |
| No activated cluster |  |  |  |  |  |  |  |
| **Bribe Conditions**  **(pooled trials in SB and DB conditions)** |  |  |  |  |  |  |  |
| ***Positive Modulation*** |  |  |  |  |  |  |  |
| SMA/MFG/CG^*^ | R | 309 | 8 | -2 | 56 | 6/24/32 | 5.32 |
| PCG/PoCG/MFG^***^ | R | 888 | 30 | -18 | 44 | 3/4/6 | 4.76 |
| FG/LG^*^ | L | 344 | -32 | -70 | -12 | 18/19/37 | 4.36 |
| MTG/MOG | L | 140 | -34 | -68 | 24 | 19 | 3.89 |
| Cuneus/Prec | L | 162 | -16 | -56 | 20 | 31 | 3.94 |
| MOG/SOG/Cuneus | R | 107 | 32 | -90 | 18 | 18/19 | 4.20 |
| Cerebellum | L | 104 | -14 | -74 | -42 |  | 4.41 |
| ***Negative Modulation*** |  |  |  |  |  |  |  |
| No activated cluster |  |  |  |  |  |  |  |
|  |  |  |  |  |  |  |  |
| **Regions encoding the relative SV**  **(pooled all conditions; GLM2b)** | | | | | | | |
| ***Positive modulation*** |  |  |  |  |  |  |  |
| MFG/SFG* | L | 300 | -22 | 24 | 40 | 8/9 | 4.59 |
| IFG | R | 115 | 50 | 26 | 18 | 46 | 4.68 |
| MeFG* | B | 314 | 2 | 48 | -14 | 10/11 | 4.50 |
| PCG/MFG/IFG | L | 173 | -36 | 12 | 28 | 6 | 4.10 |
| PCG/MFG | R | 121 | 40 | -6 | 46 | 6 | 4.62 |
| MTG/ITG*** | L | 687 | -50 | -10 | -16 | 21 | 5.06 |
| MTG/ITG*** | R | 581 | 56 | -10 | -22 | 20/21 | 5.95 |
| MTG* | R | 240 | 66 | -44 | -2 | 21/22 | 5.47 |
| MOG/SOG/AG*** | L | 2815 | -40 | -68 | 26 | 7/17/18/  19/22/39 | 5.92 |
| AG/MTG/STG/  MOG/SOG*** | R | 1149 | 36 | -88 | 16 | 7/13/19/39 | 4.82 |
| PCC/Prec/Cuneus*** | B | 2003 | -16 | -58 | 20 | 4/5/6/7/  23/30/31 | 5.39 |
| PHG/Hipp/Amyg | R | 165 | 30 | 0 | -16 |  | 5.16 |
| Cerebellum | R | 166 | 14 | -78 | -34 |  | 4.37 |
| ***Negative modulation*** |  |  |  |  |  |  |  |
| ACC | B | 124 | 14 | 28 | 20 | 32 | 4.18 |

Note: Regions shown here met the uncorrected voxel-level threshold of p < 0.001 with k = 100.

With the above uncorrected threshold as the cluster-defining threshold, clusters met the Family-Wise Error corrected cluster-level (cl-FWE) threshold were marked: ^*^p < 0.05, ^**^p < 0.01, ^***^p < 0.001

Coordinates shown here were based on Montreal Neurological Institute (MNI) coordinate system. Abbreviations: SC: Solo Control, SB: Solo Bribe, DC: Dyad Control, DB: Dyad Bribe; B: bilateral, L: left, R: right, BA: Brodmann Area; ACC: anterior cingulate cortex, AG: angular gyrus, Amyg: amygdala, CG: cingulate gyrus, FG: fusiform gyrus, Hipp: hippocampus, IFG: inferior frontal gyrus, IPL: inferior parietal lobule, ITG: inferior temporal gyrus, LG: lingual gyrus, MeFG: medial frontal gyrus, MFG: middle frontal gyrus, MOG: middle occipital gyrus, MTG: middle temporal gyrus, PCG: precentral gyrus, PHG: parahippocampal gyrus, PoCG: post-central gyrus, Prec: Precuneus, SFG: superior frontal gyrus, SMA: supplementary motor area, SOG: superior occipital gyrus, STG: superior temporal gyrus.

**Supplementary File 1h.** Neural signals during decision period (GLM1c and GLM3)

| Brain Regions | Hemisphere | Cluster Size | MNI | | | BA | T-value |
| --- | --- | --- | --- | --- | --- | --- | --- |
|  |  |  | x | y | z |  |  |
| **Neural signals during decision period**  **regardless of specific choices (N = 39, GLM1c)** | | | | | | | |
| **Main effect of Scenario:** |  |  |  |  |  |  |  |
| ***Dyad - Solo*** |  |  |  |  |  |  |  |
| LG^***^ | B | 509 | 12 | -84 | -6 | 17/18 | 4.63 |
| ***Solo - Dyad*** |  |  |  |  |  |  |  |
| No activated cluster |  |  |  |  |  |  |  |
| **Main effect of Proposer’s Conduct:** |  |  |  |  |  |  |  |
| ***Bribe - Control*** |  |  |  |  |  |  |  |
| ACC^**^ | B | 376 | 4 | 28 | 20 | 24/32 | 4.14 |
| SFG/MFG | L | 128 | -30 | 40 | 20 | 10 | 4.20 |
| Cuneus | R | 107 | 8 | -90 | 22 | 18/19 | 3.92 |
| ***Control - Bribe*** |  |  |  |  |  |  |  |
| No activated cluster |  |  |  |  |  |  |  |
| **Interaction effect** |  |  |  |  |  |  |  |
| ***(DB - DC) – (SB - SC)*** |  |  |  |  |  |  |  |
| No activated cluster |  |  |  |  |  |  |  |
| ***(SB - SC) – (DB - DC)*** |  |  |  |  |  |  |  |
| No activated cluster |  |  |  |  |  |  |  |
|  |  |  |  |  |  |  |  |
| **Choice-specific neural signals during decision period (N = 33, GLM3)** | | | | | | | |
| **Solo Scenario**  **(SB_(reject – accept)_ – SC_(reject – accept)_)** |  |  |  |  |  |  |  |
| ***Positive activation:*** |  |  |  |  |  |  |  |
| No activated cluster |  |  |  |  |  |  |  |
| ***Negative activation:*** |  |  |  |  |  |  |  |
| No activated cluster |  |  |  |  |  |  |  |
| **Dyad Scenario**  **(DB_(reject – accept)_ – DC_(reject – accept)_)** |  |  |  |  |  |  |  |
| ***Positive activation:*** |  |  |  |  |  |  |  |
| SPL/IPL/AG/Prec^**^ | R | 411 | 14 | -70 | 54 |  | 4.07 |
| MOG/IOG/FG | L | 103 | -42 | -70 | -14 | 19 | 3.93 |
| LG/FG | L | 165 | -14 | -92 | -6 | 17/18/19 | 3.59 |
| LG/FG/IOG/MOG | R | 169 | 26 | -86 | -12 |  | 3.74 |
| Cerebellum^*^ | B | 243 | 4 | -72 | -20 |  | 4.82 |
| Cerebellum/FG/MOG/IOG/ITG^**^ | R | 501 | 26 | -56 | -16 | 19/37 | 4.03 |
| ***Negative activation:*** |  |  |  |  |  |  |  |
| No activated cluster |  |  |  |  |  |  |  |
| **Main effect of Scenario:** |  |  |  |  |  |  |  |
| ***Solo*_(reject – accept)_ *- Dyad*_(reject – accept)_** |  |  |  |  |  |  |  |
| IPL/SPL/AG/SmG^†^ | R | 216 | 38 | -58 | 38 | 7/39/40 | 4.02 |
| Cerebellum | R | 126 | 2 | -74 | -18 |  | 4.67 |
| ***Dyad*_(reject – accept)_ *- Solo*_(reject – accept)_** |  |  |  |  |  |  |  |
| No activated cluster |  |  |  |  |  |  |  |

Note: Regions shown here met the uncorrected voxel-level threshold of p < 0.001 with k = 100.

With the above uncorrected threshold as the cluster-defining threshold, clusters met the Family-Wise Error corrected cluster-level (cl-FWE) threshold were marked: ^*^p < 0.05, ^**^p < 0.01, ^***^p < 0.001

Coordinates shown here were based on Montreal Neurological Institute (MNI) coordinate system. Abbreviations: SC: Solo Control, SB: Solo Bribe, DC: Dyad Control, DB: Dyad Bribe; B: bilateral, L: left, R: right, BA: Brodmann Area; ACC: anterior cingulate cortex, AG: angular gyrus, FG: fusiform gyrus, IPL: inferior parietal lobule, IOG: inferior occipital gyrus, ITG: inferior temporal gyrus, LG: lingual gyrus, MFG: middle frontal gyrus, MOG: middle occipital gyrus, Prec: Precuneus, SFG: superior frontal gyrus, SmG: supramarginal gyrus, SPL: superior parietal lobule.

**Supplementary File 1i.** PPI results with vmPFC as the seed region (PPI-GLM; N = 39)

| Brain Regions | Hemisphere | Cluster Size | MNI | | | BA | T-value |
| --- | --- | --- | --- | --- | --- | --- | --- |
|  |  |  | x | y | z |  |  |
| **Main effect of scenario:** |  |  |  |  |  |  |  |
| ***PPI: Dyad – Solo*** |  |  |  |  |  |  |  |
| MOG/Cuneus | L | 155 | -24 | -90 | 6 | 18/19 | 4.32 |
| ***PPI: Solo - Dyad*** |  |  |  |  |  |  |  |
| No activated cluster |  |  |  |  |  |  |  |
| **Main effect of proposer’s conduct:** |  |  |  |  |  |  |  |
| ***PPI: Bribe - Control*** |  |  |  |  |  |  |  |
| No activated cluster |  |  |  |  |  |  |  |
| ***PPI: Control - Bribe*** |  |  |  |  |  |  |  |
| No activated cluster |  |  |  |  |  |  |  |
| **Interaction effect** |  |  |  |  |  |  |  |
| ***PPI: (DB - DC) – (SB - SC)*** |  |  |  |  |  |  |  |
| No activated cluster |  |  |  |  |  |  |  |
| ***PPI: (SB - SC) – (DB - DC)*** |  |  |  |  |  |  |  |
| No activated cluster |  |  |  |  |  |  |  |
|  |  |  |  |  |  |  |  |
| **Individual difference analysis**  **(Regions showing enhanced**  **functional connectivity with vmPFC,**  **modulated by θ)** |  |  |  |  |  |  |  |
| **(PPI: Bribe – Control) ~ θ** |  |  |  |  |  |  |  |
| ***Positive correlation*** |  |  |  |  |  |  |  |
| No activated cluster |  |  |  |  |  |  |  |
| ***Negative correlation*** |  |  |  |  |  |  |  |
| SFG/MFG | L | 107 | -20 | 48 | -2 | 10 | 4.48 |
| SmG/AG/IPL | L | 104 | -40 | -44 | 32 | 40 | 3.92 |
| Cau/Put*** | L | 430 | -24 | 4 | 16 |  | 5.14 |
| Put* | L | 199 | -30 | -12 | -6 |  | 4.69 |
| Put/Pal* | R | 208 | 28 | -12 | -6 |  | 5.39 |
| ***Positive correlation ^a^*** |  |  |  |  |  |  |  |
| No activated cluster |  |  |  |  |  |  |  |
| ***Negative correlation ^a^*** |  |  |  |  |  |  |  |
| SFG/MFG | L | 155 | -20 | 48 | -2 | 10 | 4.65 |
| LG | L | 123 | -18 | -72 | -8 | 18/19 | 4.46 |
| Cau/Put** | L | 345 | -24 | 4 | 16 |  | 4.94 |
| Put* | L | 179 | -26 | -2 | -10 |  | 4.43 |
| Put/Pal* | R | 189 | 28 | -12 | -6 |  | 5.06 |

Note: ^a^ The control analysis also took into account the effect of ω.

Regions shown here met the uncorrected voxel-level threshold of p < 0.001 with k = 100.

With the above uncorrected threshold as the cluster-defining threshold, clusters met the Family-Wise Error corrected cluster-level (cl-FWE) threshold were marked: ^*^p < 0.05, ^**^p < 0.01, ^***^p < 0.001

Coordinates shown here were based on Montreal Neurological Institute (MNI) coordinate system. Abbreviations: SC: Solo Control, SB: Solo Bribe, DC: Dyad Control, DB: Dyad Bribe; B: bilateral, L: left, R: right, BA: Brodmann Area; AG: angular gyrus, Cau: caudate, IPL: inferior parietal lobule, LG: lingual gyrus, MFG: middle frontal gyrus, MOG: middle occipital gyrus, Pal: pallidum, Put: putamen, SFG: superior frontal gyrus, SmG: supramarginal gyrus.

**Supplementary File 1j.** Payoff stimuli used in both studies

|  |  | **Larger Payoff in the Reported Option^a^** | | | | | |
| --- | --- | --- | --- | --- | --- | --- | --- |
|  |  | **96** | **88** | **80** | **72** | **64** | **56** |
| **Offer Proportion** | **10%** | 86/10^b^ | 79/9 | 72/8 | 65/7 | 58/6 | 50/6 |
|  | **20%** | 77/19 | 70/18 | 64/16 | 58/14 | 51/13 | 45/11 |
|  | **30%** | 67/29 | 62/26 | 56/24 | 50/22 | 45/19 |  |
|  | **40%** | 58/38 | 53/35 | 48/32 | 43/29 | 38/26 |  |
|  | **50%** | 48/48 | 44/44 | 40/40 | 36/36 |  |  |
|  | **60%** | 38/58 | 35/53 | 32/48 | 29/43 |  |  |
|  | **70%** | 29/67 | 26/62 | 24/56 |  |  |  |
|  | **80%** | 19/77 | 18/70 |  |  |  |  |
|  | **90%** | 10/86 |  |  |  |  |  |

Note: ^a^ The smaller payoff always equals 100 minus the larger payoff. In the Dyad scenario, the total payoff for the proposer and the innocent third party always equals 100 (in CNY ¥), but the payoff distribution between them is reversed in two options. For example, if one option earns the proposer (the third party) 96 (4) whereas the other earns the proposer (the third party) 4 (96).

^b^In each cell, the first number refers to the payoff the proposer kept for oneself, which is calculated by the product of the larger payoff in the reported option (i.e., the payoff a proposer would like to gain) and the offer proportion. The second number refers to the amount the proposer offered the power-holder (the real participant). For example, in the first cell, the proposer reported a larger payoff (i.e., 96) and he/she offers 10% out of the payoff to the participant (i.e., 10), so that he/she could keep the remaining amount for him-/herself (i.e., 86). For the sake of simplicity, all numbers are rounded up to the nearest integers. All these payoff distributions were presented once in each of the four conditions. Notably, we did not adopt all the possible distributions (see the missing cells), as we assumed that the proposer should be rational so that after the offer proposition he/she should still earn more than the other option with a smaller payoff.

**Supplementary File 1k.** Bivariate correlation between payoff-related parametric modulators (PM) used in GLM1a and GLM1b

|  | Expected gains for the proposer | Expected gains for the participant |
| --- | --- | --- |
| Expected gains for the participant | -0.83^***^ |  |
| Expected losses to the third party^a^ | 0.01 | 0.55^***^ |

Note: *Pearson* correlation coefficients are reported in the table.

^a^ According to the design, this variable was calculated by the following equation: larger payoff in the reported option – (100 - larger payoff in the reported option).

Significance: ^***^*p* < 0.001
